# Molecular Dynamics Simulation of the Thermal Behavior of Hydroxyapatite

**DOI:** 10.3390/nano12234244

**Published:** 2022-11-29

**Authors:** Ilya Likhachev, Nikolay Balabaev, Vladimir Bystrov, Ekaterina Paramonova, Leon Avakyan, Natalia Bulina

**Affiliations:** 1 Institute of Mathematical Problems of Biology, Keldysh Institute of Applied Mathematics, RAS, 142290 Pushchino, Russia; 2 Physics Faculty, Southern Federal University, 344090 Rostov-on-Don, Russia; 3 Institute of Solid State Chemistry and Mechanochemistry, Siberian Branch, Russian Academy of Sciences, 630128 Novosibirsk, Russia

**Keywords:** hydroxyapatite, modeling, molecular dynamics simulation, structural defects, OH group and OH vacancy, OH evaporation, thermal behavior, nanomaterials

## Abstract

Hydroxyapatite (HAP) is the main mineral component of bones and teeth. Due to its biocompatibility, HAP is widely used in medicine as a filler that replaces parts of lost bone and as an implant coating that promotes new bone growth. The modeling and calculations of the structure and properties of HAP showed that various structural defects have a significant effect on the properties of the material. By varying these structural heterogeneities, it is possible to increase the biocompatibility of HAP. An important role here is played by OH group vacancies, which are easily formed when these hydroxyl groups leave OH channels of HAP. In this case, the temperature dependence of the concentration of OH ions, which also determines the thermal behavior of HAP, is important. To study the evaporation of OH ions from HAP structures with increasing temperatures, molecular dynamics simulation (MDS) methods were used in this work. As a program for MDS modeling, we used the PUMA-CUDA software package. The initial structure of HAP, consisting of 4 × 4 × 2 = 32 unit cells of the hexagonal HAP phase, surrounded by a 15-Å layer of water was used in the modelling. Multiple and statistically processed MDS, running calculations in the range of 700–1400 K, showed that active evaporation of OH ions begins at the temperature of 1150 K. The analysis of the obtained results in comparison with those available in the literature data shows that these values are very close to the experiments. Thus, this MDS approach demonstrates its effective applicability and shows good results in the study of the thermal behavior of HAP.

## 1. Introduction

Hydroxyapatite (HAP) is the main mineral component of bones (about 50% of total bone mass) and teeth (96% in enamel). Due to its natural biocompatibility, hydroxyapatite is used in medicine as a filler that replaces parts of the lost bone (in traumatology and orthopedics, hand surgery), and as an implant coating that promotes the growth of new bone [1,2,3,4,5,6,7,8]. In dentistry, hydroxyapatite is used in toothpastes as a component that remineralizes and strengthens tooth enamel. Currently, HAP is used in both photocatalysis [9] and photoluminescence [10], which makes HAP a promising material for cell imaging and drug release monitoring.

The modeling and calculations of the structure and properties of HAP showed that many of the properties of real HAP samples are determined by the presence of various structural defects in it (oxygen vacancies as well as whole OH hydroxyl groups, insertions, and substitutions of various atoms in the structure of HAP) [7,8,11,12]. Among them, an important role is played by OH group vacancies, which are easily formed when these hydroxyl groups leave the internal structure of the HAP OH-channel. This is determined by the features of non-covalent interactions and the molecular-like internal configuration of HAP with tubular channels, which consist of chains of hydroxyl OH groups linked by hydrogen bonds [6,7,8,9]. An important point here is also the thermal stability of HAP [13,14,15], which strongly depends on the concentrations of the OH groups into the channels at different temperatures.

Here it should be noted once again, that the basic structure of HAP has such specific features as extended structural channels formed by a chains of the OH groups (OH-channels) [5,6,7,8,9,16,17,18,19].

There is the possibility of proton movement along these channels [20,21,22,23] (including, under certain conditions, even tunneling through potential barriers along this OH channel [21]) and the appearance of defects such as vacancies of protons, oxygen, and whole OH groups [7,8,9,11,12,20,21,22,23,24,25,26]. Various defects have different effects on the change in the properties of a regular HAP periodic lattice. Therefore, for example, the HAP samples during fabrication and treatment are heated to certain temperatures (700–900 K and above [13,14,15,22,23,24,25]). It is known that OH groups under these conditions leave the samples. The especially intense escape of OH from HAP begins at temperatures of about 1073 K [25] or 1173 K [13,15]. As a result, HAP is dehydrated [7,13,25,26]. During subsequent cooling, OH groups from the environment are reintroduced into these channels, but their concentration depends on the humidity of the environment and the OH vacancies partially can remain in the HAP samples after cooling in air [4,6,7,8,13,14,25,26].

Another specific and important defect in HAP are oxygen vacancies [4,6,7,8,9,11,12,27,28]. They can arise from both the OH group and from the PO_4_ group. Moreover, oxygen vacancies can be of different types depending on the position of the oxygen atom and the symmetry of the given atomic PO_4_ group [7,12]. Their formation is possible at higher temperatures (1300–1500 K) [13,14,15,24,25,26,27,28] or also under radiation exposure [29].

In addition, complex mixed defects, such as the “O vacancy + OH vacancy” type, can also occur [7,8,9,11,12]. Recently, all these defects have been studied in sufficient detail by modern computer methods, and their photoelectronic, photocatalytic, and photoluminescent properties have also been confirmed experimentally [9,10].

In addition to the oxygen and hydroxyl vacancies, HAP can contain defects such as interstitials and ion substitutions [7,8,11,12,30,31,32,33,34,35,36]. Various modern experimental and theoretical methods are now used to study them. Among them, computer modeling and calculations, including first principles ab initio calculations, density functional theory (DFT) methods, and quantum-chemical semi-empirical methods [5,6,7,8,9,37,38,39,40,41,42,43,44,45,46,47,48,49], are the most important. These methods that are implemented in various software tools [50,51,52,53,54], constantly are developed, allowing them to be effectively used in calculations and many computational studies.

However, these approaches are well developed mainly for the study of equilibrium and stable system states under constant external conditions. The study of dynamic changes in such complex structures is still a rather complex computational problem.

One of the important modern numerical methods for studying the properties of various complex atomic and molecular structures is precisely the method of molecular dynamics simulation (MDS) [55,56,57]. The MDS method consists of solving Newton’s equation of motion for an atomic–molecular system with a certain time step, by repeatedly calculating the forces acting on each atom, and then using those forces to update the position and velocity of each atom in the next step. This leads to obtaining 3-dimensional spatial trajectories for all atoms in the system. Many properties of the system can be calculated from these atomic trajectories.

The goal of the computer simulation of molecular systems is to calculate macroscopic behavior based on microscopic interactions. Comparing models and trajectories made under different conditions, one can determine the influence of a wide variety of factors causing atomic and molecular perturbations, including the influence of temperature and the thermal behavior of the structures under study with its increase.

One of the main problems of MDS is the use of a sufficiently accurate function of the interaction energies for the atomic–molecular system under study. Within the framework of classical MDS [56], interaction methods based on classical molecular mechanics (MM) are used here—these are the so-called “force fields” (FF) approaches [56,57,58,59,60]. These forces in classical MD simulations are calculated using a model known as the MM force field, which is consistent with various quantum mechanical calculation results and usually with a set of experimental measurements. For example, a typical FF includes elements that describe electrostatic (Coulomb) interactions between atoms, elements like springs that model the preferred length of each covalent bond, various angles, and elements that describe several other types of interatomic interactions. Among such force fields widely used in practice are well known ones such as Amber, GROMAX, CHARMM, etc., [58,59,60,61]. However, all such force fields are inherently approximate. Nevertheless, comparisons of simulations with various experimental data show that force fields have improved significantly over the past decade [56,61], especially in combination with modern multiprocessor computing systems and advanced methods of parallel computing. For example, in work [57], the full-atomistic classical MDS of the laser heating of silicon dioxide thin films in the range up to a temperature of 1000 K and 2000 K is performed. All simulations were carried out using the GROMACS program [59,62].

In our case, at the first stage, it is important to assess, overall, the behavior of the entire HAP structure with easily mobile OH groups depending on temperature. For this purpose, we applied the approaches of well-established classical MDS [56], using known force fields, such as Amber [58]. In this study, we use the approach based on the software complex PUMA-CUDA, developed in our Molecular Dynamics Laboratory IMPB RAS [63,64,65,66], which has a high performance due to the use of various parallel programming technologies (parallel operation on multiprocessor systems with shared memory, distributed memory, and also operation on graphics accelerators). These calculations were carried out using a K-60 hybrid supercomputer installed at the KIAM RAS Shared Use Center [67]. In this study, we consider thermal processes occurring in HAP structures in detail based on classical MDS methods, developed with original in-house algorithms.

## 2. Computational Details, Main Models, and Methods

### 2.1. Main Methods and Used Software

To analyze statistical data and processing of several MDS run series at different temperatures, we developed a special algorithm. Temperature dependence of the OH concentration (inside and outside of HAP sample) and the thermal stability of HAP up to ~1500 K are very important [13]. In this regard, the resulting trajectories of MD were investigated by the specially developed method implemented in the software (IMPB RAS, Pushchino, Russia) “Trajectory Analyzer of Molecular Dynamics” (TAMD) developed in the Molecular Dynamics Laboratory of IMPB [68,69,70].

The PUMA-CUDA software package (IMPB RAS, Pushchino, Russia), whose physics is based on the PUMA software package [63,64,65,66], was used as an MDS program. PUMA-CUDA supports operation and simulation of systems based on constant number N, constant volume V, and constant temperature T (NVT), as well as constant pressure P (NPT) ensembled in periodic boundary conditions. Additionally, it has a high performance due to the use of various parallel programming technologies (parallel operation on multiprocessor systems with shared memory, distributed memory, and also operation on graphics accelerators).

The AMBER99 [71,72] force field as well as the TIP3P water model were used [73]. The TIP3P water model specifies a 3-site rigid water molecule with charges and Lennard-Jones parameters assigned to each of the 3 atoms [74,75]. The resulting trajectories of molecular dynamics were investigated by the TAMD [69,70].

### 2.2. Initial Structural Data

One of the common structural peculiarities of HAP is connected with the pseudo-one-dimensional character of the structure. OH^−^ ions form a long chain along the main structural *c* axis [7,8,9,16,17,18,19,20,21], which is often named an ‘OH-channel’ in HAP. The HAP general formula is Ca_10_(PO_4_)_6_(OH)_2_, where the hydroxyl units show stochastic orientation along OH channels.

The structure of HAP is primarily based on its initial pristine stoichiometric structural phase—hexagonal P6_3_, with a unit cell consisting of 44 atoms and containing structural OH channels with 2 hydroxyl OH groups in each elementary unit cell [4,6,7,8,9,16,21,22,23,24]. Depending on the orientation of these OH groups, the cells can have different symmetry groups: P6_3_/m—for the hexagonal disordered phase (when the orientation of the OH groups is random) and P6_3_—for the hexagonal ordered phase (when the orientation of the OH groups is parallel and directed in the same direction, which creates its own internal polarization [7,8,36].

**Figure 1:**
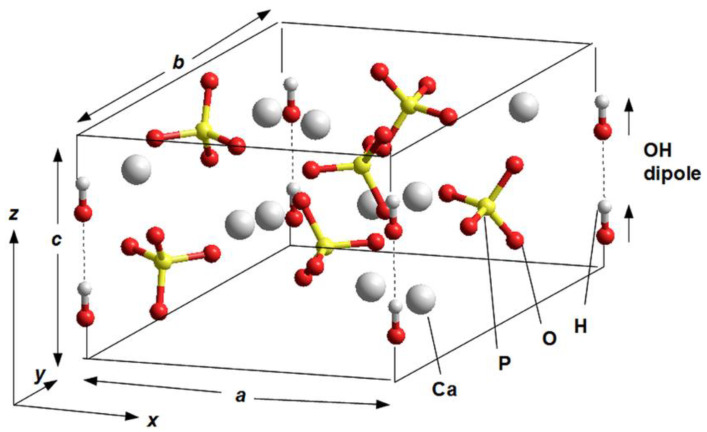
One unit cell model of the Hydroxyapatite (HAP: Ca_10_(PO_4_)_6_(OH)_2_)—in P6_3_ hexagonal phase. All OH groups are oriented in the same direction. They are positioned at the four corners of the unit cell, but only one pair in one corner belongs to this elementary unit cell; the other three pairs belong to neighboring unit cells (here the atoms are color-marked: gray—Ca, red—O, yellow—P, white—H). (Adapted with permission from ref. [8]; IOP Publishing, 2015).

**Figure 2:**
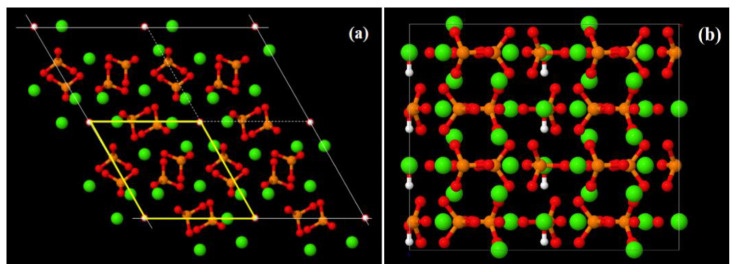
Super-cell 2 × 2 × 2 = 8 HAP unit cell model: (**a**) in *c*-axis projection—perpendicular to OH-channel (see in center), the unit cell is shown by yellow lines; (**b**) in b-axis projection—the OH groups in vertical line positions for the P6_3_ phase.

**Figure 3:**
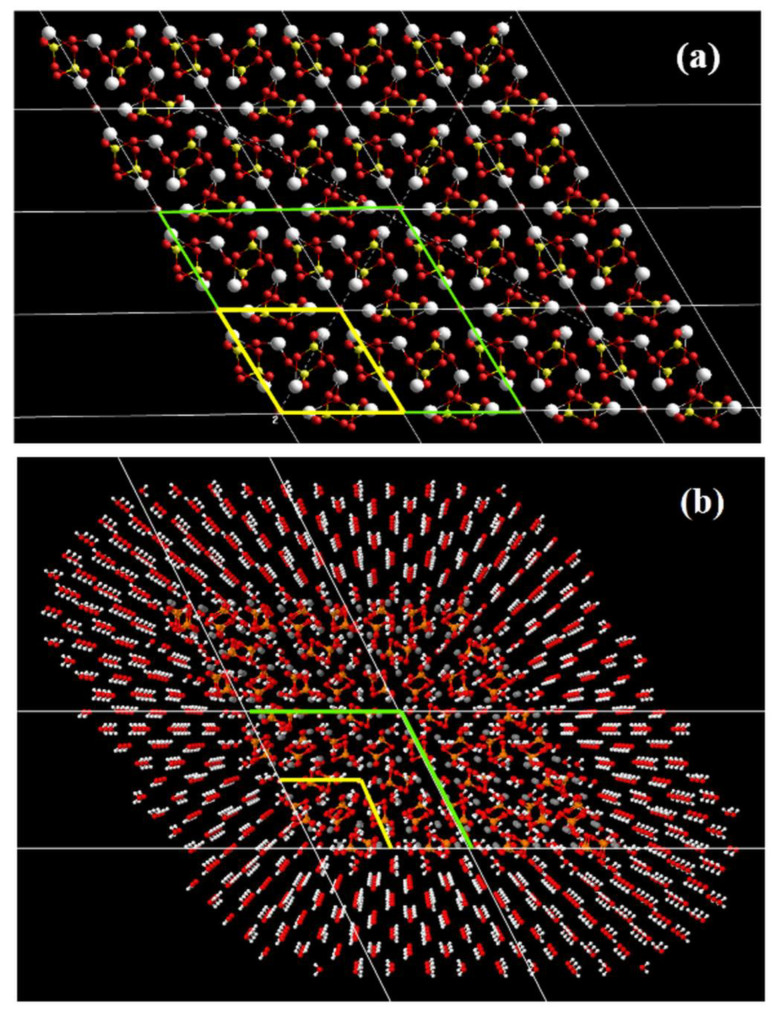
Images of large HAP32 supercell models with 4 × 4 × 2 = 32 unit cell of HAP and 1408 atoms: (**a**) top view—along the c axis of the OH channel (a yellow line highlights the one unit cell and green lines show one 2 × 2 × 2 supercell with OH-channel in the center); (**b**) big HAP supercell model in isometric projection from c-axis, surrounded by water molecule layers with 1504 water molecules.

Some earlier calculations were performed using one elementary unit cell model of hexagonal P6_3_ HAP [7,8] (Figure 1), with parameters *a* = *b* = 9.417 Å, *c* = 6.875 Å [17]. The main peculiarity of the next study is the introduction of the supercells model made up of 2 × 2 × 2 = 8 HAP unit cells with 352 atoms (space group P6_3_) for hexagonal HAP phase [7,11,12] (Figure 2). In the present work, in order to carry out MDS runs at different temperatures and study the evaporation of OH ions from the HAP crystal, a new model of the HAP structure, consisting of 4 × 4 × 2 = 32 HAP unit cells (1408 atoms) as a large super-cell model or large HAP32 cluster, was constructed using PUMA software package [63,64,65] (Figure 3).

The structure of this HAP32 cluster (Figure 3a) was surrounded by a 15-Å layer of water with 1504 water molecules (Figure 3b). The size of the calculated HAP cell with water was 53.40 Å × 31.94 Å × 13.87 Å, which was determined in the NPT ensemble. The simulation was carried out under periodic boundary conditions with a computational cell size of ~76.49 Å × 57.37 Å × 38.24 Å in an NVT ensemble at a constant temperature maintained by a collisional thermostat. Each dimension of the computational cell was increased by ~25 Å. It is necessary for water molecules’ relaxation in the biggest volume during heating. Note that water plays an important role, simulating the environment into which OH groups fly out, exerting a different effect on OH at different temperatures as the steam from the media.

To construct a water environment for modeling under periodic boundary conditions, usually all available space (minus the molecules of the desired system) is filled with water. But when it comes to temperatures well above the boiling point, it’s wise to leave some space free for steam simulation. This steam turned out to be quite saturated, although less saturated than when boiling. We are only interested in the fact of separation of molecules and ions from the main system. It makes no sense to simulate a long free path of vapor molecules, since it will only increase the amount of computation. We only investigate the fact of detachment of molecules, and not what happens to them in the future. They came off the supercell—it means they flew out of the environment, far from the hydroxyapatite surface.

To create initial equilibrium conditions for conducting MDS, the entire model structure of a HAP32 cluster of 4 × 4 × 2 = 32 HAP hexagonal unit cells, prepared in this way in the PUMA-CUDA program, surrounded by 1504 water molecules, was relaxed for about 500 ps = 0.5 ns at T = 300 K. At this temperature, water began to look like usual water, and not like a regular structure. Nothing happened with the HAP cell; it behaved like a solid at room temperature.

To carry out the MDS of the described HAP system at different temperatures, two main different approaches were applied: (1) relaxation of the system at selected constant temperatures and (2) linear heating of the system.

At the same time, to perform a statistical analysis of sets of different MDS runs at different temperatures, 16 realizations were selected in each of the approaches obtained from the initial (prepared) data of the system, which relaxed within 10 ns independently of each other at different temperatures.

The energy of interatomic interactions was calculated within the modified force field AMBER99, using necessary parameters for 9–6 and 6–12 Lennard-Jones potentials [71].

### 2.3. Simulation Procedure

Thus, the heated area is a cell with the size 76.49 Å × 57.37 Å × 38.24 Å, inside which there is an HAP cluster of 4 × 4 × 2 = 32 elementary HAP cells, surrounded by 1504 water modules in the initial layer 15 A thick (Figure 3b). Two types of numerical MDS runs were carried out:

(1) with a successive series of fixed temperatures from 800 to 1175 K in steps of 25 K, which relaxed within 10 ns independently of each other at these temperatures; 16 implementations obtained from the initial data for the entire system (HAP32, prepared as described above) were carried out;

(2) during linear heating of this entire system (HAP32 cluster with a surrounding set of water molecules) at a rate of 100 K/ns (or 0.1 K/ps) by changing the temperature T_Ref_ of the collisional thermostat, in the temperature range from 700 to 1400 K with a total heating time up to 7 ns (16 independent MDS runs also).

The temperature is taken into account in the model in the same manner as in the method of all-atom molecular dynamics, with the use of a collisional thermostat, where the medium in which the modeled system occurs is simulated by point particles demonstrating the Maxwell velocity distribution [63,64,65]. The velocity distribution corresponds to a certain temperature T_Ref_. At random times, virtual particles of the medium elastically collide with the particles of the system. The motion equations have the form:

(1) $$m_{i}\frac{dv_{i}}{dt}=F_{i}+\sum_{k}f_{ik}\cdot \delta \left(t-t_{ik}\right),$$

(2) $$F_{i}=\frac{-dU}{dx_{i}},$$

Here, δ(t) is the Dirac delta function, and $f_{ik}$ is a stochastic source of force leading to jumps of the i-th atom velocity at random times $t_{ik}$. The value of the velocity jump is calculated as a result of collision of two point particles which have the velocities v (for a modeled particle) and v_0_ (for a virtual particle) before the collision:

(3) $$\Delta v\left(t\right)=\frac{2m_{0}}{m_{0}+m}\left(v_{0}\left(t\right)-v\left(t\right)\right),$$

Here, m is the mass of a modeled particle, and m_0_ is the mass of a virtual particle. The velocities v_0_ are chosen from the Maxwell distribution. This equation follows from the law of conservation of energy and momentum.

### 2.4. Algorithm for Simulation Procedure Analysis

To analyze the simulation results, the Trajectory Analyzer of Molecular Dynamics (TAMD) was used with an added algorithm for counting ejected ions, which is described below. 

1. A zero vector V (0, 0, …, 0) of dimension n is constructed, where n is the number of OH ions in the system. If the i-th element is equal to 0, then this means that the i-th OH ion is connected to the system; if an ion leaves the system, the i-th element is 1. 

2. At each step of the trajectory (which corresponds to 1 ps), the minimum distances from each OH ion to the remaining hydroxyapatite atoms are calculated. If the distance between the OH ion with the serial number i is greater than 5 Å, then we equate the corresponding component of the vector V(i) = 1 to one.

3. Also, at each step, we calculate the sum of the components of the vector V. This will be the number of detached OH ions to this point in time.

4. We build the dependence of the number of detached OH ions on time. Such an algorithm makes it possible to count the number of ions that have ever detached up to a given point in time.

### 2.5. Evaluation of the Energy of the HAP System upon Removal of OH Groups

To compare the energy costs for the detachment of OH ions from the HAP cluster during thermal heating with the results of MDS runs at different temperatures, we also evaluated the change in the energy of the system upon the detachment and removal of individual OH groups from the HAP cluster.

To calculate a change in the total energy of the HAP32 cluster system upon the removal of OH groups from the system over a long distance and to estimate the energy of detachment and removal of an individual OH ion, the molecular mechanics (MM) force field AMBER method included in the HyperChem software [53] was used here. Calculations of changes in the total energy of the system were carried out for each distance *z* between the surface of the HAP32 cluster and the position of the removed OH ion for the cases of different numbers of OH groups and when they were removed from different OH- channel. The model HAP32 (Figure 3a) without water molecules surrounding the cluster, but in lateral projection along the *b* axis, was used for this calculation. The calculation scheme for the model in this projection is given below in Section 3.3.

### 2.6. Evaluation of the Dynamics of HAP Behavior at Different Temperatures

To estimate the possible temperature ranges in which the stable configurations of HAP model systems during MDS runs exist, such MDS runs on the HAP cluster model, without surrounding it with a medium of water molecules, were preliminarily carried out. This also made it possible to determine the temperatures when the transformation, destruction, or melting of HAP model systems will occur, when calculating it using the AMBER99 force field methods (that is, MM methods at each point of the performed MDS run). For this, a HAP cluster was taken, similar to HAP32 (Figure 3a), but with a larger size of 16 × 4 × 2 = 128 HAP unit cells (HAP128, consisting of 5465 atoms). This system of such a large number of atoms (at the limiting level of the applied computational methods and tools) also allowed us to evaluate the stability and accuracy of the procedure proposed by the MDS of the PUMA-CUDA software and the correctness of the calculations. Accordingly, first, we carried out MDS runs for the model of HAP cluster HAP128 with 16 × 4 × 2 unit cells at heating temperatures up to 2100 K without water molecules surrounding it. Eight independent MD experiments were carried out on the relaxation of the system at constant temperatures: 700, 900, 1100, 1300, 1500, 1700, 1900, 2100 K.

## 3. Results and Discussion

### 3.1. Dynamics of HAP Model Behavior and Melting at Different Temperatures

**Figure 4:**
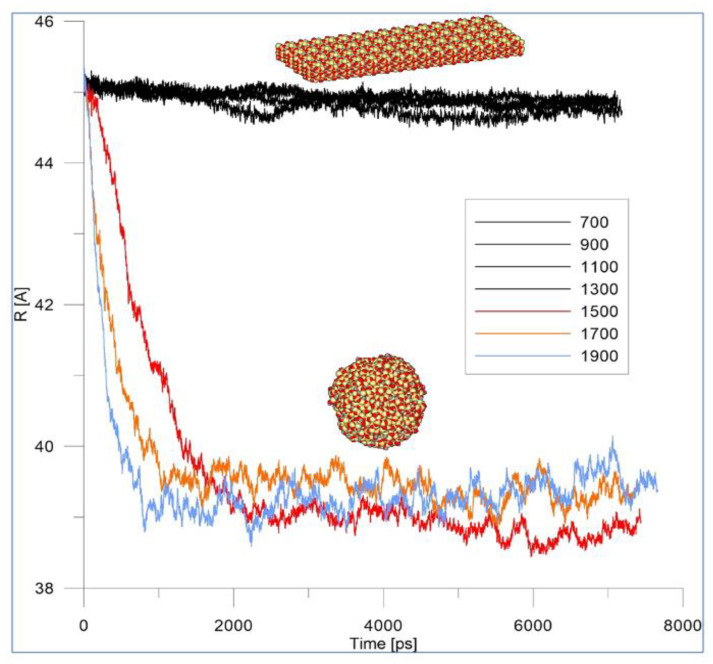
The dependence of the radius of gyration on time for HAP128 cluster of 16 × 4 × 2 supercells.

Thus, after preliminary MDS runs on the HAP128 model (without surrounding it with water molecules) at various temperatures T, indicated above in Section 2.6, the following results were obtained (Figure 4).

The melting of HAP on the MD trajectory was visually observed as a transformation of a flat structure into a coil (coil-shaped, globulation) one. For a quantitative assessment of the melting dynamics, the dependence of the radius of gyration on time is plotted and tested. The radius of gyration is such a value, the square of which, when multiplied by the mass of the body, will be equal to the moment of inertia of the body about the axis. This value can characterize a change in the shape of the body. At low temperatures, fluctuations in the radius of gyration are practically indistinguishable (black curves in Figure 4). With an increase in temperatures from 1500 K and above, the radius of gyration rapidly decreases (colour curves in Figure 4). 

The rate of initial fall of the curved lines in Figure 4 (3 colored curves) increases with increasing temperature. Thus, it was found that at temperatures of 1500 K and above, the HAP cluster actually collapses (decomposes) or melts. This result corresponds to the experimentally observed data that, when heated above 1500 K HAP, decomposes or changes the phase [13,14,15]. 

Therefore, later (below) in this work, when modeling the processes of emission (evaporation) of OH groups from HAP studied by MDS, we considered the temperature range 700 K to 1400 K. We chose the initial temperature of 700 K, since at this temperature it is known that the process of evaporation of OH groups from HAP does not occur [13,14,15].

### 3.2. Detachment of OH Ions at Heating

(1) *Results of the MDS run at the fixed temperatures*.

**Figure 5:**
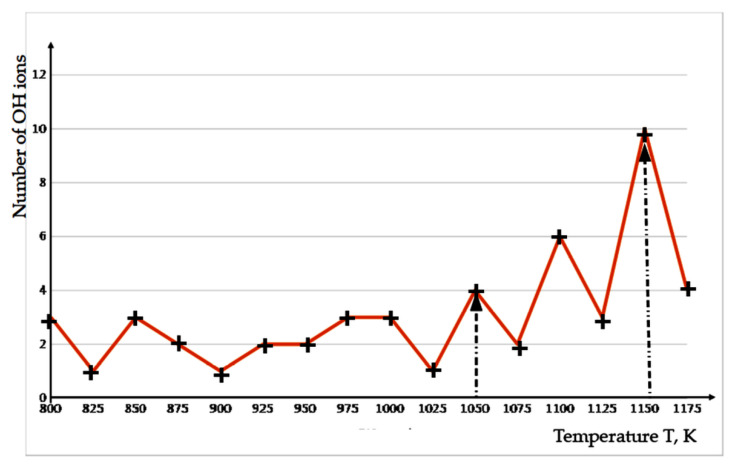
The detached OH ions number during 10 ns relaxation of MDS run at each constant temperature T (the results of the 16 independent MDS runs are shown). The crosses mark the values of the number of detached OH ions at each fixed T, and the line shows their conditional change; 1-st arrow marks the temperature of the beginning of the intensive release of OH ions from the HAP32 cluster; 2-nd arrow marks the temperature with 10 OH ions departure.

Figure 5 shows an example of changing the number of detached OH groups from the HAP32 cluster at different constant temperatures (from 800 to 1175 K in steps of 25 K) during 10 ns MDS run relaxation in a water environment independently for each of these temperatures. Thus, 16 independent MDS runs of the entire system were repeated, and showed various data depending on each fixed temperature. Based on the data, we can conclude that the middle evaporation of 1–3 OH ions is observed in the interval of 800–1025 K. With a further increase in temperature, the dehydroxylation process intensifies. Thus, at 1150 K, a detachment of 10 OH groups is already observed.

(2) *Results of the MDS run with linear temperature growth*.

**Figure 6:**
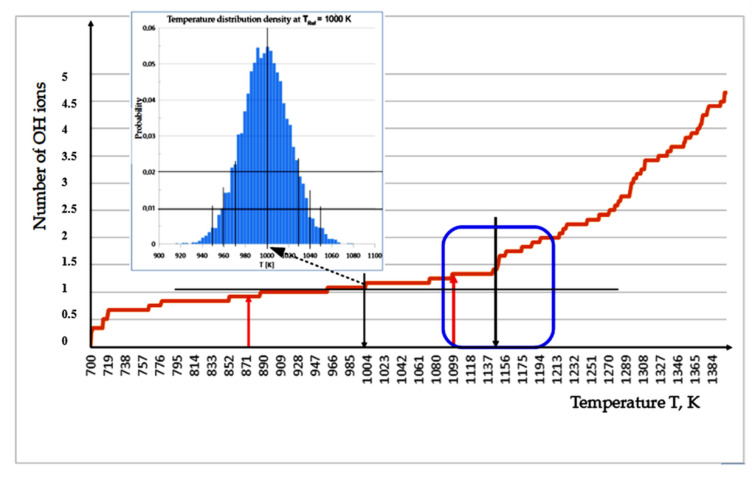
Temperature dependence of the average number of detached OH ions in MDS run at linear heating with rate 0.1 K/ps (the result of averaging over 16 MDS runs is shown). The black arrows mark the characteristic temperatures when the number of escaped OH ions from the HAP32 cluster is changed; the red arrows show the corresponding temperatures obtained from the MM calculations of the change in the total energy of the system (see details in Section 3.3). The inset shows the temperature distribution around the T_Ref_ of the used MDS thermostat. The temperature range of the beginning of the active departure of OH ions is outlined by the blue rectangle.

The system of HAP32 cluster in the water environment was heated at a rate of 100 K/ns (or 0.1 K/ps) by changing the temperature T_Ref_ of the collisional thermostat. This temperature determinates, according to relation T_Ref_ = 700 K + 0.1 K/ps × t (here t is the time of modeling in [ps]). The 16 independent realizations of heating MD run experiments were done (Video S1 shows an example of one of these MD runs). Averaging over all the experiments gives the dependence shown in Figure 6.

Based on Figure 6, we can conclude that, at heating with rate 0.1 K/ps, the active evaporation of OH ions begins from a temperature of about 1150 K (it is shown by the black arrow in Figure 6).

The indication of this temperature T*_exit_~1150 K can serve as an estimate from above, but not from below. It is impossible to find a specific value for the evaporation temperature, because for such small systems, the average temperature has a spread of several tens of Kelvin. As is known, the distribution of velocities of molecules corresponds to Maxwell’s law. If one can wait indefinitely, until there will definitely be OH ions on the outside of the system, the kinetic energy of which will be greater than the energy sufficient to overcome the non-valent bonds that hold the ion in the system. In other words, the process of evaporation of OH ions is quite possible at lower temperatures, on the order of T*~800–850 K (shown by the black arrow in Figure 6). We are limited only by the duration of the MD experiment. If one waits an arbitrarily long time (and this is already microseconds), then, perhaps, the evaporation process can be observed even at 300–400 K. That is, everything is determined by the probability of the process.

The obtained data is confirmed by the experimentally detected changes in the HAP structure upon heating and keeping the samples at certain temperatures [13,14,15]. Therefore, taking into account the thermodynamic definition of the concept of temperature and distribution statistics in a collisional thermostat during the MDS process, it can be seen that the temperature distribution, for example, around T_Ref_ at T = 1000 K has such a Gaussian form, shown in the insertion in Figure 6.

It is possible to carry out here a comparative assessment of the Maxwell and Gauss distributions and the corresponding temperatures. As a result, one can obtain a relation connecting the statistical dispersion σ and thermostat temperature T_Ref_: 2σ^2^ = T_Ref_, and therefore σ = (T_ref_/2)^1/2^.

In this case, for each temperature, the following dispersion s can be determined: for T_Ref_ = 1000 K, σ = ~22.36 K~22 K; T^*^_exit_ = 1150 K, σ* = ~23.98 K~24 K. That is, the difference between these dispersions is insignificant. As one knows, the dispersion of 1σ covers 68% of all values around the average value; that is, here we can take T^*^_exit_ = 1150 ± 24 K. At the same time, while 2σ is already 95% of all values, there are already T^*^_exit_ = 1150 ± 48 K, while T_Ref_ = 1000 ± 44 K.

Therefore, it is very close between these values. For the case of 3σ, all values overlap within the interval ± 3σ with a probability of 99.7%. Thus, for 3σ we have T^*^_exit_ = 1150 ± 66 K. This data is very comparable with data from Table 1 below.

This makes it possible to estimate the temperature range of the onset of intense escape of OH ions. On average, it can be assumed that the spread or dispersion of temperatures at these values can be estimated approximately as the interval ΔT~±44 K (or ±66 K). Thus, we can conclude that the evaporation of OH ions (namely, the detachment of OH ions from the HAP cluster and their removal to a sufficiently large distance), which we have established using the MDS method, begins actively from temperatures T*_exit_ = 1150 ± 44 K (or T^*^_exit_ = 1150 ± 66 K).

We can say that the temperature range, when the active departure (exit) of OH ions from the HAP sample is already counted, is in the range from 1100 to 1200 K. But this process can also start in a wider range, starting from 850–873 K. Everything depends on taking into account the probability and holding time (relaxation or annealing).

**Figure 7:**
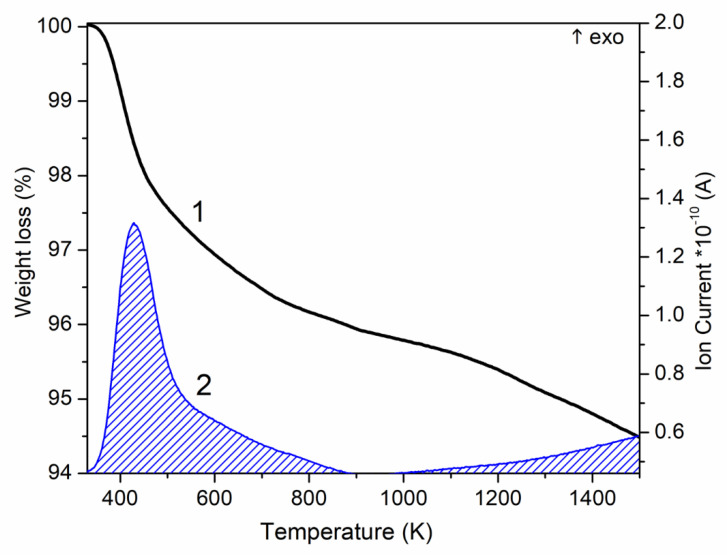
Thermal analysis of the HA powder: 1—weight loss; 2—evolved water (from [13]).

The data obtained using multiple MDS runs for this OH ion active departure at temperature T*_exit_ = 1150 K are well consistent with many known data [13,14,15,25]. First of all, we would like to emphasize the obvious similarity of the obtained curve in Figure 6 with the thermal analysis data given in the paper by Bulina [13] (see in Figure 7). It is also consistent with the thermogravimetric analysis data from the work [15], where average temperature values are very close for the dehydroxylation of HAP and the removal of water molecules in the air atmosphere (or OH ions in water vapor for our case) starts at 900 °C = 1173 K. At lower temperatures, adsorbed and lattice water (if it presents in the material) is released by heating HAP experimentally.

Experimental work [13] informs that the dehydroxylation process in HAP probably starts at a lower temperature of 600 °C and continues up to temperatures above 1300 °C (Figure 7). In our case, as can be seen from the curve line graphs in Figure 6, this process starts from (850–873) K and can continue up to 1500 K, when the transformation (decomposition) of the HAP structure is already taking place; that is, it directly corresponds to the data shown in Figure 7 (but in the Kelvin temperature scale). As noted also in [13], the amount of released water increases with an increase in temperature up to 1200 °C and hardly changes at high temperatures; this is consistent with our data as well.

### 3.3. Changes of the Energy of the HAP System upon Removal of OH Groups

**Figure 8:**
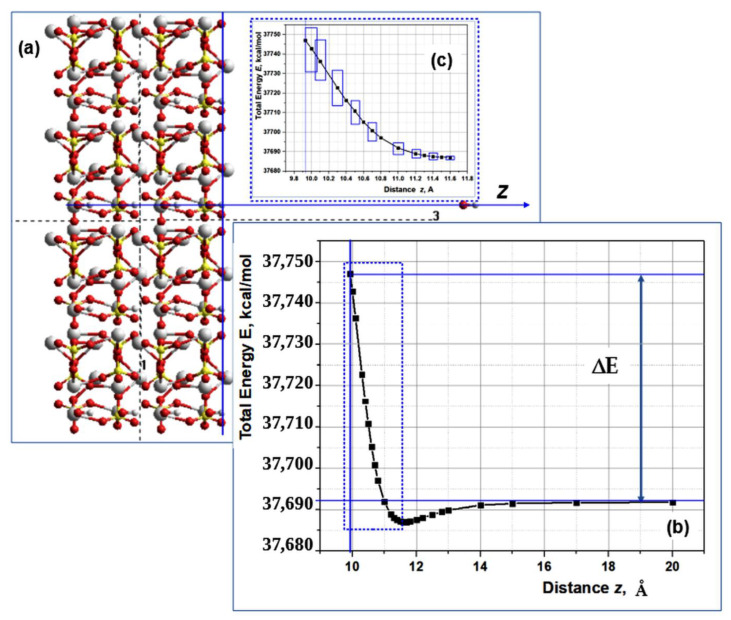
Model of OH going out from HAP32 and change of the system total energy: (**a**) the distance *z* is along *c*-axis of the HAP32 cluster model, which is shown as axis number 3 (it is perpendicular to the surface plane of the HAP32 cluster); axis number 1 is along *a*-axis of the HAP32 model; axis number 2 is along *b*-axis of the HAP32 cluster (it is perpendicular to the image plane); (**b**) the graph shows the profile of the change in the total energy of the systems as the OH ion moves away from the surface of the HAP32 cluster (an example of one typical calculation); (**c**) the inset shows the selected part (outlined by a blue rectangle with a short dashed line).

In order to better understand the processes taking place here, we also carried out calculations to estimate the change in the energy of a large HAP cluster when OH ions escape from it and move away over long distances. The energy of detachment and removal of OH groups from the HAP32 cluster have been calculated. Here, we made use of our HAP32 model (with 4 × 4 × 2 = 32 HAP unit cells) and the MM method with the similar AMBER force field option, but in the HyperChem software package [53], for the system energy calculation at every position of the detached OH ions. The schema of the considered model, presented in Figure 8, as well as the typical total energy profile are shown.

In these calculations, we considered several different cases of models: first, when the one OH group is detached from the HAP32 cluster and removed by a large distance; second, when 2 OH groups (in different configurations—from one OH channel and from their two adjacent OH channels) are detached and removed from the HAP32 cluster; and, third, when 3 OH groups are detached and removed from the HAP32 cluster.

The obtained values of changes in the total energy |ΔE|, as well as the energy change |ΔE_1_| per one cell of the HAP32, are summarized in Table 1. Here, the temperature T values, corresponding in terms of thermal energy (according to relation T = |ΔE_1_|/k_B_, where k_B_ = 8.6173·10^−5^ eV/K is the Boltzmann constant) to these processes of detachment and removal of the various OH groups, is also provided.

As can be seen, the simultaneous removal of three OH ions at once over a long distance (100 Å or more angstroms) requires a noticeable increase in thermal energy compared to the removal of one or two OH ions.

**Table 1:** Change in energy when extracting OH groups from a HAP32 cluster of 4 × 4 × 2 = 32 unit cells when they are removed to a large distance (more than 100 Å).

	Model	Total Energy Change |ΔE|, kcal/mol	Energy Change per 1 Unit Cell |ΔE_1_|, kcal/mol	Energy Change per 1 Unit Cell |ΔE_1_|, eV	Corresponding Temperature T=|ΔE_1_|/k_B_, K
1	HAP32—1_OH (OH from one OH-channel)	55.21 ± 5.37	1.725 ± 0.167	0.075 ± 0.007	870 ± 84
2	HAP32—2_OH (from one OH-channel)	69.33 ± 6.73	2.167 ± 0.210	0.094 ± 0.009	1090 ± 106
3	HAP32—2_OH (from two OH-channel)	70.62 ± 6.85	2.207 ± 0.214	0.096 ± 0.009	1114 ± 108
4	HAP32—3_OH (from one OH-channel)	101.13 ± 9.81	3.160 ± 0.307	0.137 ± 0.013	1590 ± 154

*Error analysis of the energy change when OH groups are removed from HAP.* Although the mathematically calculated values of the energies of the system (according to the classical relations of the Amber MM method used in this work) unambiguously correspond to precisely specified atomic coordinates, physically the atomic coordinates cannot be absolutely specified exactly. Errors in the coordinates of atoms here inevitably arise due to quantum effects and the influence of temperature. First, they have some “smearing” due to the Heisenberg uncertainty principle, which becomes especially important at small sizes. For the z-coordinate, momentum *p,* and Planck’s constant *h*: Δz·Δ*p* = h/2π; that is, Δz = *h*/2πΔ*p* is the uncertainty of the z-coordinate. Using energy relation E = (Δ*p*)^2^/2*m*, where m is the particle mass, one can express Δ*p* = (2*m*E)^1/2^. Secondly, thermal energy is expressed as E_T_ = k_B_T, where k_B_ is Boltzmann’s constant. As the temperature increases, so does the thermal energy and the vibrations of atoms associated with it in the crystal lattice. This also leads to errors in the accuracy of specifying the coordinates of atoms, which leads to an error in the calculations of the determined energy of the system. Thus, Δ*p* = (2*m*k_B_T)^1/2^ and Δz = (h/2π)/(2*m*k_B_T)^1/2^.

Let us make an estimate for the case of the hydrogen atom (as part of the OH group). Considering that the reduced Planck constant h/2π = 1.0545718·10^−34^ J·s, the mass of a hydrogen atom *m* = 1.67·10^−27^ kg, and the thermal energy at T = 1000 K will be about E_T_ = 0.08617 eV = 0.1379·10^−19^ J, we get Δz~1.554·10^−11^ m = 0.1554 Å~0.2 Å.

The error in calculating the energy will then be determined here by the change in energy at such distances Δz~0.2 Å.

The greatest changes in energy at such small Δz distances, corresponding to the error in the coordinate z, occur near the HAP surface itself. This is clearly seen in Figure 8b. It also shows the size of energy and coordinate errors in the form of rectangles. The energy errors (corresponding to Δz) calculated in this way and having a maximum near the HAP surface itself turn out to be of the order of Δ(E_01_) = ~10.73 kcal/mol, when changing z from z_0_ = ~9.9 Å to z_1_ = 10.1 Å (for Δz = 0.2 Å).

In this case, the total error in the calculation of the entire change in the total energy of the system when OH is removed to a large distance is determined precisely by this energy error at the very surface of the HAP, since when OH groups are removed to a large distance, the total energy of the system actually does not change (Figure 8b). With a long distance (~100 Å) of one OH group along the axis of the central OH channel, this change is |ΔE|~55.2143 kcal/mol~55.21 kcal/mol (Figure 8 and Table 1). Taking into account the total error, the corresponding spread of the values will be Δ(|ΔE|)~±5.37 kcal/mol around an average value of |ΔE|~55.21 kcal/mol, and this gives a relative error of about δ~5.37/55.2143 = ~0.097; that is, of about 9.7%~10%. The corresponding values with errors when recalculating the energy per one HAP unit cell are |ΔE_1_|~1.725 ± 0.167 kcal/mol = 0.075 ± 0.007 eV, and for corresponding T = 870 ± 84 K.

With this in mind, we have similarly estimated the errors for other values in Table 1 and have now indicated them in Table 1. As one moves away from the HAP surface, the error in the energy values decreases, as can be clearly seen in Figure 8c. At large distances z~20–100 Å, the energy itself changes little (as can be seen from the graph in Figure 8b). The relative error in this case decreases to the value of d~0.00815, i.e., ~0.8%, i.e., does not exceed 1%.

On the curve plot presented above in Figure 6, we marked the temperature values obtained during detachment and removal of one OH ion (at temperature T~870 K) and two OH ions (at temperature T~1090 K) with red arrows. As can be seen, these temperatures are in good agreement with other data on the initial values of the evaporation temperature of individual OH ions and on the beginning of active mass OH groups evaporation (dehydroxylation) of HAP. At the same time, it should be noted that the simultaneous emission of 3 OH groups and their removal over a long distance already requires much more energy and the corresponding temperature increases to T~1590 K, which is close to the decomposition of the HAP structure.

It should be noted that in these models and calculations, we did not take into account the influence of the water molecule (surrounding the HAP32 cluster), which, of course, will change the trajectories of OH ions in the collisions. Accounting for this effect should lead to a shift in the average temperature T of these processes.

It should also be noted that the obtained temperatures are consistent with the work of Magsunaga, who indicated that HAP begins to lose water, producing a significant amount of OH defects at temperatures up to 1073 K, and stoichiometric HAP itself can no longer be stable above 1073 K [25].

The study of changes in the structure and properties of HAP during heating is actively carried out both experimentally and by calculation methods, including density functional theory (DFT) methods [7,8,9,11,12,36,37,38,39,40,41,42,43,44,45,46,47,48,49]. The formation of the OH vacancies significantly affects the properties of HAP [7,9,11].

Moreover, it has been established that at high temperatures, various structural rearrangements are possible here with the transition of HAP to oxyapatite (OAP) (Ca_10_(PO_4_)_6_O), or the mixture of TCP (Ca_3_(PO_4_)_2_) and CaO [13]. But in this work, we aimed specifically at studying the possibility and application of MDS to study the process of dehydroxilation in HAP during heating. These other points are beyond the scope of this work.

Nevertheless, we would like to note the important role of the DFT in the study of OH vacancies in HAP [7,8,9,11]. A deeper understanding of the mechanisms (and processes) of the formation of defects in HAP such as OH group vacancies is given here using the DFT approaches [40,41,42,43,44,45,46,47,48,49].

**Figure 9:**
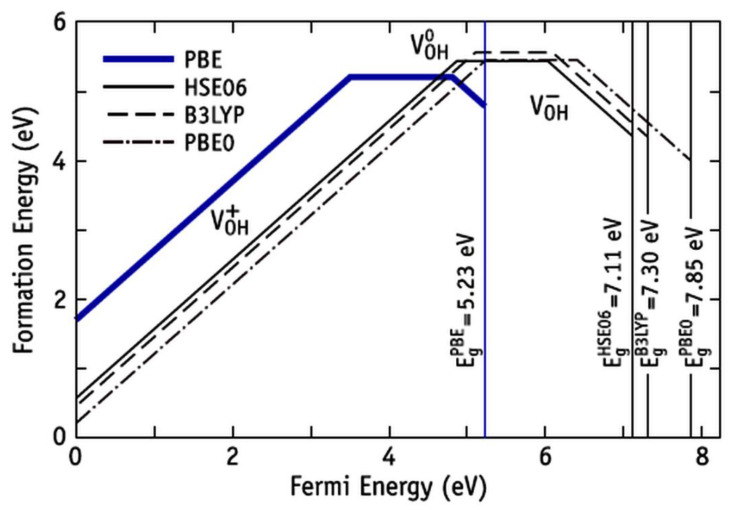
Calculated formation energy of V_OH_ in HAP as a function of the Fermi energy, using different approximations to the DFT exchange–correlation interactions. Positive, neutral, and negative charge states are represented by lines with positive, zero, and negative slope, respectively. For each functional, the Fermi energy can vary between the valence band top (at *E*_F_ = 0 eV) and the respective conduction band minimum (*E*_F_ = *E*g). (Adapted with permission from ref. [11]; AIP Publishing, 2015).

Recently, detailed calculations of the formation of OH vacancies with HAP have been carried out [7,11,12] and a complex dependence of the energy formation of an OH vacancy in HAP on the charge state of the HAP crystal, as well as on the calculation method used by the DFT, has been established. Such calculations showed that for a neutral OH vacancy, the energy of formation with the use of the PBE functional turns out to be of the order of 5 eV, while for the hybrid functionals HSE and B3LYP, it reaches ~5.5 eV value [11] (Figure 9).

At the same time, the latest DFT calculations for the case of 2 OH groups vacancies show that the energy of their formation is on the order of ~10 eV; these data agree in order with the experimental value for the cases of γ- and β-TCPs. There are, of course, differences associated with a difference of 1 calcium atom. But fundamentally here is the fact that these are completely dehydrated structures that do not have OH groups. We present the relevant data in Table 2.

The energies are calculated using a hybrid DFT approach as described in [11]. The values reported in Table 2 are measured in eV per six PO_4_ units to ease the comparison between materials with slightly different stoichiometry.

These results obtained using DFT approaches clearly show the physical reason and difference of the different quantity of the OH vacancy arising in the HAP structure for various cases and conditions.

However, all of these DFT calculations consider the “fixed”, equilibrium, and stable states for structures that have already been formed after any impacts and exist under certain conditions—that is, this structure is already the final product. For example, as a result of heating HAP to a certain temperature, when already in such an initial stoichiometric HAP, structural changes occurred as a result of heating and HAP was no longer HAP, but was transformed into a HAP-2OH structure or into OAP, or TCP (depending on the final conditions of the heating process and annealing).

The proximity of formation energies of TCP and HAP-2OH (“HAP without OH”) shows the possibility of spontaneous transition between these phases. Our recent calculations show a slight benefit of the second phase over the first one, which is not confirmed experimentally. The reason is that we consider dense polymorph of TCP (γ-TCP) more convenient for the calculations since it has fully occupied crystallographic positions. More reliable is the transition from OAP to less dense TCP polymorphs. Among the phases without hydrogen and OH, the OAP is more favorable than TCP or HAP-2OH. We consider this question in another work.

As mentioned above, the very dynamic of such structural changes (such as changes in the HAP structure upon heating) is a rather complex process. Simulation of such changes in HAP and their dynamic description cannot yet be sufficiently carried out rigorously and adequately using currently used quantum methods, including DFT. The development of MDS in the direction of mastering the approaches of precise quantum MDS is only just beginning to take place.

However, it is possible that with further development of approaches and methods of quantum MDS, this will still be achieved, including the analysis of the dynamics of changes in HAP structures upon heating.

## 4. Conclusions

The thermal behavior of the HAP structure during heating processes was studied in this work. As a result of our work, we have established that the process of departure (detachment and removal) of OH groups from HAP is described quite well by the proposed MDS procedure. This calculation was carried out in the framework of the classical MDS, using the Amber force field, implemented into PUMA-CUDA software, and developed in IMPB RAS. The main temperatures described the evolution of the OH groups departure from HAP during heating, and those obtained in this MDS are in line with experimentally known data. The comparable estimation of the thermal energies for these temperatures using molecular mechanics Amber methods (in HyperChem software) show a good match between these energies and temperatures. It should also be noted here that the dispersion or errors obtained of values around the average values of temperatures T turn out to be of the same order of magnitude obtained above by different methods—from the estimation of the dispersion of a collisional thermostat (usually used in MD runs) and from the calculations of energies when OH groups are removed from the HAP surface by molecular mechanics methods using Amber force fields. This increases the reliability of the results of the work.

The DFT calculations of the formation of one OH and two OH vacancies in the HAP structure show relative rise of this energy with HAP structural changing, corresponding to experimental data. One of the important points to note here is that the obtained simulation results can be useful to experimenters, since they show new possible effects and predict the quantitative values of the quantities that can be measured. In particular, the formation energy of HAP without one and two OH groups, as well as OAP, which are close to the values for known measurements using examples such as γ-TCP, but are not yet identified well in experimental studies.

Further studies aimed at the development of quantum MDS for modeling and dynamical studies of the HAP heating processes and its thermal behavior will be planned in future.

## Figures and Tables

**Table 2:** Formation energy of hydroxyl group vacancies V_1OH_ and V_2OH_ in HAP. (From Kohn-Sham formalism of DFT calculation basically described in work [11,12]).

	Calculation Using Method Reported in [11]		Experimental Data [76]	
Compound	Δ_f_*H*, eV/(PO_4_)_6_	|ΔE|, eV/(PO_4_)_6_	Δ_f_*H*, eV/(PO_4_)_6_	|ΔE|, eV/(PO_4_)_6_
HAP	−128.49	0	−138.88	0
γ-TCP	−117.54	10.95	−128.48	10.40
β-TCP	−117.38	11.11		
HAP-1OH (without 1OH)	(−122.99) *	(5.0–5.5) *		
HAP-2OH (without 2OH)	−117.68	10.81		
OAP	−123.16	5.33		

* Data based on Figure 9 from [11].

## Supplementary Materials

The following supporting information can be downloaded at: https://www.mdpi.com/article/10.3390/nano12234244/s1. Video S1: The heating process of HAP.
